# Supplementary material for: MetaQTL: a package of new computational methods for the meta-analysis of QTL mapping experiments
Source: BMC Bioinformatics. 2007 Feb 8;8:49. doi: 10.1186/1471-2105-8-49 (PMC1808479; doi:10.1186/1471-2105-8-49)
Supplement: Additional File 7 — MetaQTL Package : jar file and tutorial. This Zip archive contains both the MetaQTL JAR file and the files of the tutorial. [file 1471-2105-8-49-S7.zip › org.inra.metaqtl/doc/index-files/index-16.html]

Q-Index


|  |  |  |
| --- | --- | --- |
| **Overview** \| Package \| Class \| Use \| **Tree** \| **Deprecated** \| **Index** \| **Help** | |  |
| **PREV LETTER**   **NEXT LETTER** | **FRAMES**    **NO FRAMES**     **All Classes** |  |


A B C D E F G H I K L M N O P Q R S T U V W X Y Z 

---


## **Q**

**Qtl** - Class in org.metaqtl: A QTL (Quantitative Trait Loci) **Qtl()** - Constructor for class org.metaqtl.Qtl: **QTL** - Class in org.thalia.bio.entity: Class Description Here **QTL()** - Constructor for class org.thalia.bio.entity.QTL: **QTL(String, IBioEntity)** - Constructor for class org.thalia.bio.entity.QTL: **QTL** - Static variable in interface org.thalia.bio.IBioLocus: **Qtl.QTLCross** - Class in org.metaqtl: This class stores the informations on the cross design used to detect the QTL. **Qtl.QTLCross()** - Constructor for class org.metaqtl.Qtl.QTLCross: **Qtl.QTLProj** - Class in org.metaqtl: This class stores the QTL projection properties. **Qtl.QTLProj()** - Constructor for class org.metaqtl.Qtl.QTLProj: **Qtl.QTLStats** - Class in org.metaqtl: This class stores the usual statistics for the QTL. **Qtl.QTLStats()** - Constructor for class org.metaqtl.Qtl.QTLStats: **Qtl.QTLSupport** - Class in org.metaqtl: This class stores information on the QTL Confidence Interval **Qtl.QTLSupport()** - Constructor for class org.metaqtl.Qtl.QTLSupport: **Qtl.QTLTrait** - Class in org.metaqtl: This class stores the trait description of a QTL **Qtl.QTLTrait()** - Constructor for class org.metaqtl.Qtl.QTLTrait: **QTL\_CI\_FROM** - Static variable in interface org.metaqtl.IBioLocusProperties: The key for the QTL confidence interval lower boundary. **QTL\_CI\_LOD\_DECREASE** - Static variable in interface org.metaqtl.IBioLocusProperties: The key for the QTL confidence interval LOD decrease. **QTL\_CI\_TO** - Static variable in interface org.metaqtl.IBioLocusProperties: THe key for the QTL confidence interval upper boundary. **QTL\_CI\_WIDTH** - Static variable in class org.metaqtl.graph.MetaGraphPar: **QTL\_CI\_WIDTH\_CEX** - Static variable in class org.metaqtl.graph.MetaGraphPar: **QTL\_CROSS\_NAME** - Static variable in interface org.metaqtl.IBioLocusProperties: The key for the QTL cross name. **QTL\_CROSS\_SIZE** - Static variable in interface org.metaqtl.IBioLocusProperties: The key for the QTL cross size. **QTL\_CROSS\_TYPE** - Static variable in interface org.metaqtl.IBioLocusProperties: The key for the QTL cross type. **QTL\_HSPACE** - Static variable in class org.metaqtl.graph.MetaGraphPar: The horizontal space between Qtl shapes. **QTL\_NAME\_COLOR** - Static variable in class org.metaqtl.graph.MetaGraphPar: The color of the QTL names. **QTL\_NAME\_FONT** - Static variable in class org.metaqtl.graph.MetaGraphPar: The font used for the Qtl names. **QTL\_NAME\_SPACE** - Static variable in class org.metaqtl.graph.MetaGraphPar: **QTL\_NAME\_VSPACE\_CEX** - Static variable in class org.metaqtl.graph.MetaGraphPar: The space magnification between the Qtl name and the Qtl confidence interval. **QTL\_PALETTE** - Static variable in class org.metaqtl.graph.MetaGraphPar: The color palete for the QTL partition. **QTL\_PCH\_SIZE** - Static variable in class org.metaqtl.graph.MetaGraphPar: **QTL\_POS\_HEIGHT\_CEX** - Static variable in class org.metaqtl.graph.MetaGraphPar: **QTL\_POS\_WIDTH\_CEX** - Static variable in class org.metaqtl.graph.MetaGraphPar: **QTL\_PROB\_WIDTH** - Static variable in class org.metaqtl.graph.MetaGraphPar: **QTL\_PROJ\_INTSCALE** - Static variable in interface org.metaqtl.IBioLocusProperties: The key for the QTL flanking marker interval projection rate. **QTL\_PROJ\_MAPSCALE** - Static variable in interface org.metaqtl.IBioLocusProperties: The key for the QTL confidence interval projection rate. **QTL\_PROJ\_ORIGIN** - Static variable in interface org.metaqtl.IBioLocusProperties: The key for the map of origin of the QTL. **QTL\_PROJ\_SHAREFLANKING** - Static variable in interface org.metaqtl.IBioLocusProperties: The key for the QTL flanking marker interval order preservation. **QTL\_PROJ\_SWAPFLANKING** - Static variable in interface org.metaqtl.IBioLocusProperties: The key for the QTL flanking marker interval reverse order. **QTL\_STATS\_ADDITIVE** - Static variable in interface org.metaqtl.IBioLocusProperties: The key for the QTL additive effect. **QTL\_STATS\_DOMINANCE** - Static variable in interface org.metaqtl.IBioLocusProperties: The key for the QTL dominance effect. **QTL\_STATS\_RSQUARE** - Static variable in interface org.metaqtl.IBioLocusProperties: The key for the QTL rsquare. **QTL\_TICK\_WIDTH\_CEX** - Static variable in class org.metaqtl.graph.MetaGraphPar: The magnification for the tick of the QTL. **QTL\_TRAIT\_GROUP** - Static variable in interface org.metaqtl.IBioLocusProperties: The key for the QTL trait group. **QTL\_TRAIT\_NAME** - Static variable in interface org.metaqtl.IBioLocusProperties: The key for the QTL trait name. **QTL\_TRAIT\_UNIT** - Static variable in interface org.metaqtl.IBioLocusProperties: The key for the QTL trait unit. **QTL\_TREE\_SCALE** - Static variable in class org.metaqtl.graph.MetaGraphPar: **QTL\_TREE\_SPACE** - Static variable in class org.metaqtl.graph.MetaGraphPar: **QTL\_VSPACE** - Static variable in class org.metaqtl.graph.MetaGraphPar: The vertical space between Qtl shapes. **QtlAdapter** - Class in org.metaqtl.adapter: This class defines some usefull methods to adapt `Qtl` object into other kinds of objects or the inverse. **QtlAdapter()** - Constructor for class org.metaqtl.adapter.QtlAdapter: **qtlByChrom** - Variable in class org.metaqtl.MetaQtlAnalysis: The matrix of qtl by chromosome **qtlCI** - Variable in class org.metaqtl.QtlPartition: The Qtl confidence intervals. **QTLClust** - Class in org.metaqtl.main: **QTLClust()** - Constructor for class org.metaqtl.main.QTLClust: **QtlClustAlgorithm** - Class in org.metaqtl.algo: This class defines methods to perform a QTL meta-analysis on a genetic map by applying a clustering of QTL based on a gaussian mixture model and the EM-algorihtm. **QtlClustAlgorithm(IBioGenome, IBioOntology)** - Constructor for class org.metaqtl.algo.QtlClustAlgorithm: **QtlClustAlgorithm(IBioGenome, String, IBioOntology)** - Constructor for class org.metaqtl.algo.QtlClustAlgorithm: **QTLClustInfo** - Class in org.metaqtl.main: **QTLClustInfo()** - Constructor for class org.metaqtl.main.QTLClustInfo: **qtlIdx** - Variable in class org.metaqtl.MetaQtlResult: The indices of QTL which are implied in the analysis. **QtlLayer** - Class in org.metaqtl.graph: **QtlLayer(double, double)** - Constructor for class org.metaqtl.graph.QtlLayer: **QtlLayerBuffer** - Class in org.metaqtl.graph: **QtlLayerBuffer()** - Constructor for class org.metaqtl.graph.QtlLayerBuffer: **QTLLOCUS** - Static variable in interface org.thalia.bio.IBioEntityTypes: **QTLModel** - Class in org.metaqtl.main: **QTLModel()** - Constructor for class org.metaqtl.main.QTLModel: **qtlNames** - Variable in class org.metaqtl.QtlPartition: The qtl names **qtlPart** - Variable in class org.metaqtl.graph.ChromGraph: The Qtl partition **QtlPartition** - Class in org.metaqtl: **QtlPartition()** - Constructor for class org.metaqtl.QtlPartition: **QtlPartitionAdapter** - Class in org.metaqtl.adapter: This class defines some methods to adapt complient objects into a `QtlPartition` object. **QtlPartitionAdapter()** - Constructor for class org.metaqtl.adapter.QtlPartitionAdapter: **QtlPartitionLayer** - Class in org.metaqtl.graph: **QtlPartitionLayer(double, double)** - Constructor for class org.metaqtl.graph.QtlPartitionLayer: **qtlPos** - Variable in class org.metaqtl.QtlPartition: The Qtl positions **QTLProj** - Class in org.metaqtl.main: **QTLProj()** - Constructor for class org.metaqtl.main.QTLProj: **QtlProjAlgorithm** - Class in org.metaqtl.algo: This class defines some methods to project QTL positions from a genetic map to another. **QtlProjAlgorithm(IBioGenome[], IBioGenome, IBioLocus[])** - Constructor for class org.metaqtl.algo.QtlProjAlgorithm: Creates a new instance of `QtlProjAlgorithm` for the given configuration of projection. **qtls** - Variable in class org.metaqtl.Chromosome: The QTLs located on this chromosome. **QtlShape** - Class in org.metaqtl.graph: **QtlShape()** - Constructor for class org.metaqtl.graph.QtlShape: **qtlShapes** - Variable in class org.metaqtl.graph.QtlLayer: The Qtl shapes. **QTLTree** - Class in org.metaqtl.main: **QTLTree()** - Constructor for class org.metaqtl.main.QTLTree: **QtlTreeAlgorithm** - Class in org.metaqtl.algo: This class defines methods to perform agglomerative hierarchical QTL clustering. **QtlTreeAlgorithm(IBioGenome, IBioOntology)** - Constructor for class org.metaqtl.algo.QtlTreeAlgorithm: **QtlTreeLayer** - Class in org.metaqtl.graph: **QtlTreeLayer(double, double)** - Constructor for class org.metaqtl.graph.QtlTreeLayer: **QtlUnit** - Class in org.metaqtl.graph: **QtlUnit()** - Constructor for class org.metaqtl.graph.QtlUnit

---


|  |  |  |
| --- | --- | --- |
| **Overview** \| Package \| Class \| Use \| **Tree** \| **Deprecated** \| **Index** \| **Help** | |  |
| **PREV LETTER**   **NEXT LETTER** | **FRAMES**    **NO FRAMES**     **All Classes** |  |


A B C D E F G H I K L M N O P Q R S T U V W X Y Z 

---
